# Epidemiology of and programmatic response to tuberculosis in Solomon Islands: analysis of surveillance data, 2016–2022

**DOI:** 10.5365/wpsar.2024.15.1.1106

**Published:** 2024-03-06

**Authors:** Manami Yanagawa, Ben Gwali, Henry Kako, Noel Itogo, Lia Tanabose, Fukushi Morishita

**Affiliations:** a World Health Organization Regional Office for the Western Pacific, Manila, Philippines.; b National Tuberculosis Programme, Ministry of Health and Medical Services, Honiara, Solomon Islands.; c World Health Organization Representative Office for Solomon Islands, Honiara, Solomon Islands.; † Deceased.

## Abstract

**Objective:**

To identify progress and challenges in the national response to tuberculosis (TB) in Solomon Islands through an epidemiological overview of TB in the country.

**Methods:**

A descriptive analysis was conducted using the national TB surveillance data for 2016–2022. Case notifications, testing data, treatment outcomes and screening activities were analysed.

**Results:**

The number of case notifications was 343 in 2022, with an average annual reduction of the case notification rate between 2016 and 2022 of 4.7%. The highest case notification rate was reported by Honiara City Council (126/100 000 population) in 2022. The number of people with presumptive TB tested by Xpert^®^ rapidly increased from zero in 2016 to 870 in 2022. Treatment success rate remained consistently high between 2016 and 2022, ranging from 92% to 96%. Screening for HIV and diabetes mellitus (DM) among TB patients in 2022 was 14% and 38%, respectively. Most patients (97%) were hospitalized during the intensive phase of treatment in 2022; in contrast, during the continuation phase, the proportion of patients treated at the community level increased from 1% in 2016 to 63% in 2022. Despite an increase in household contact investigations, from 381 in 2016 to 707 in 2021, the uptake of TB preventive treatment (TPT) was minimal (7% among eligible child contacts).

**Discussion:**

This epidemiological analysis in Solomon Islands reveals both notable achievements and challenges in the country’s TB programme. One major achievement is a potential actual reduction in TB incidence. Challenges identified were potential underdetection of cases in rural areas, suboptimal community-based care, and insufficient contact tracing and uptake of TPT. It is crucial to address these challenges (e.g. by optimizing resources) to advance the national TB response.

Tuberculosis (TB) is one of the most important infectious diseases globally, with an estimated 10.6 million people developing the disease and 1.6 million people dying from it in 2021. (*1*) The coronavirus disease (COVID-19) pandemic greatly disrupted TB case detection, resulting in a reduction in global case notifications of 18% in 2020 and 10% in 2021. (*2*)

Solomon Islands was classified as a country with upper-moderate TB incidence in 2021. (*3*) Passive case finding is the primary method of case finding; it is supplemented by systematic screening, including contact investigation and cross-referral of people with suspected TB from clinics for HIV and diabetes mellitus (DM). Xpert^®^ MTB/RIF has been used as a primary TB diagnostic test, and there were nine sites using Xpert in 2023. If Xpert testing is unavailable, sputum smear tests are used. TB treatment is centralized either at the National Referral Hospital or provincial hospitals, with a minimum of 2 months of hospitalization required during the intensive phase. After discharge, treatment for the continuation phase is provided, with the support of the clinic nearest to the patient’s residence. Patients can opt for facility-based ambulatory care, community-based care or self-administration of TB medicines.

The national TB response is guided by the Tuberculosis National Strategic Plan 2021–2023. (*4*) Various activities have been planned and implemented to detect, treat and prevent TB, with considerable financial support from international donors.

This paper describes the epidemiology of TB in Solomon Islands, and highlights programmatic achievements and challenges through the analysis of national surveillance data collected by the National TB Programme (NTP). Beyond addressing the country’s concerns, this analysis contributes valuable insights to the broader understanding of TB challenges in other island countries with similar conditions.

## Methods

This descriptive analysis included the trend of TB case notifications, age and sex distributions, type of diagnosis, laboratory data, treatment outcomes, HIV screening, DM screening, contact investigation, TB preventive treatment (TPT) and mode of care. Data were sourced from quarterly TB reports submitted by provincial TB coordinators to the NTP. Province-level analysis was performed for the case notifications, treatment outcomes, and HIV and DM screening.

In Solomon Islands, case information is recorded on the TB patient registry at the provincial hospital, and the area or rural health centre. The information is compiled into both aggregated and case-based Microsoft Excel^®^ reports by a provincial TB coordinator on a quarterly basis. Laboratory data are reported from the Chronic Cough Register. The quarterly reports are then submitted to the NTP for programme monitoring and evaluation.

The definitions of cases and treatment outcomes are in accordance with the World Health Organization (WHO) reporting framework for TB. (*5*) Data analysis and visualization were conducted with the statistical software package R-4.3.1 (Comprehensive R Archive Network: https://cran.r-project.org/).

## Results

### TB case notifications

**Fig. 1:**
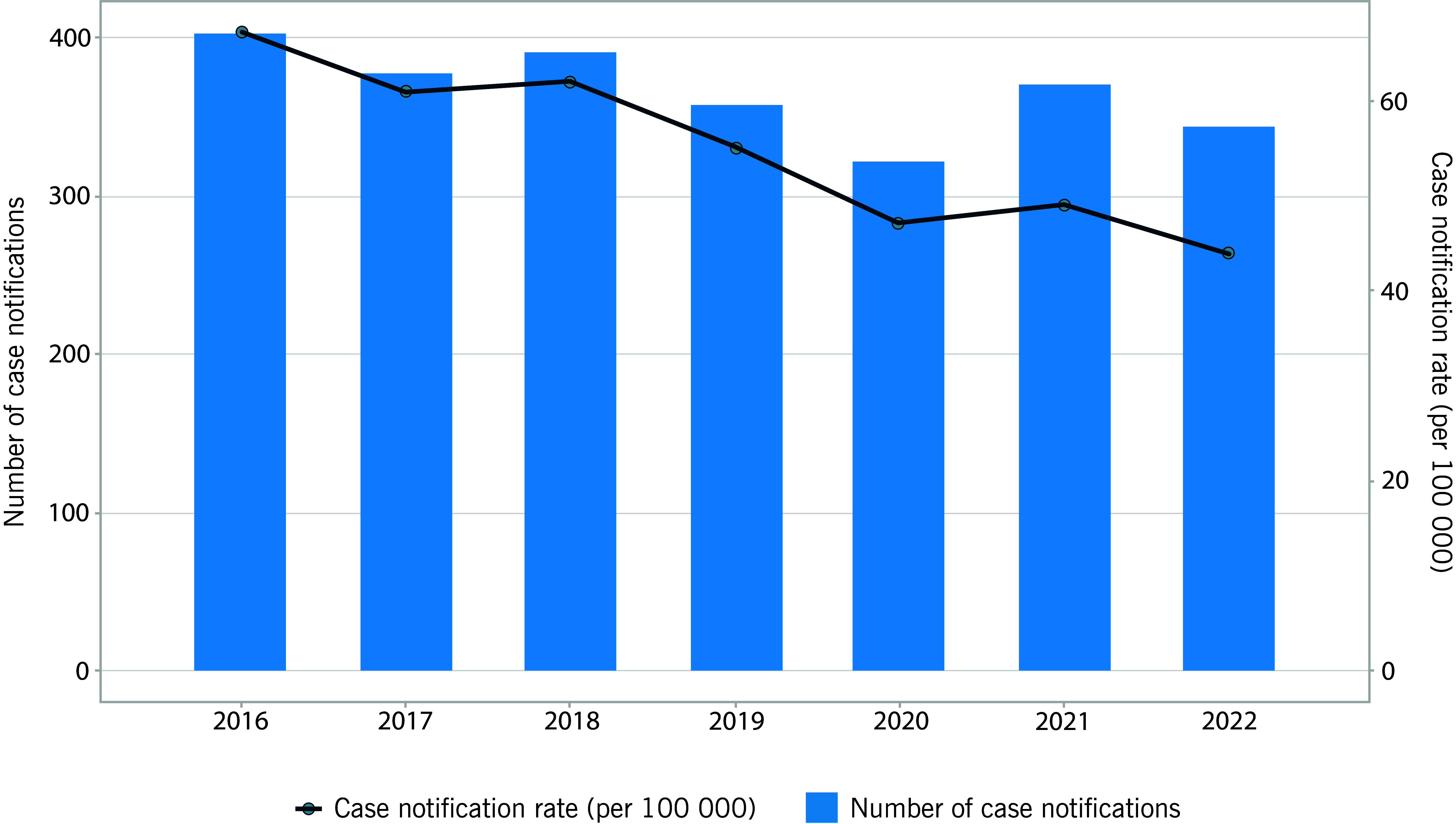
Number of TB notifications and rate per 100 000 population, Solomon Islands, 2016–2022

**Fig. 2:**
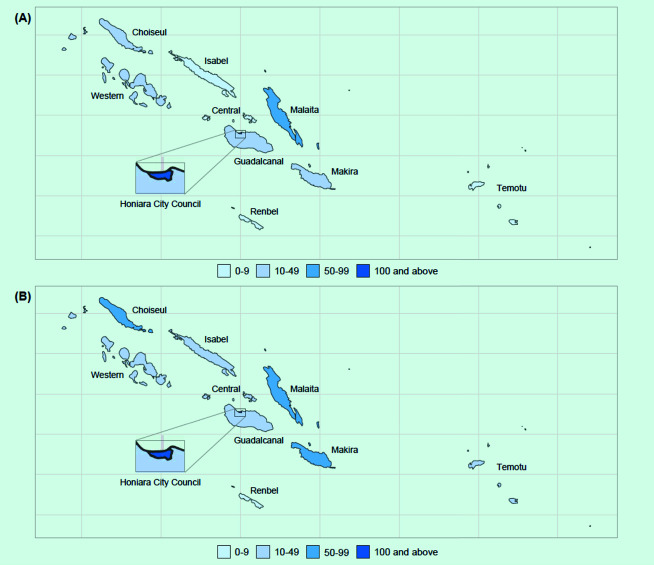
Number of TB notifications (A) and rate per 100 000 population (B) by province, Solomon Islands, 2022

In 2022, there were 343 cases and the rate of case notifications was 44 per 100 000 population (**Fig. 1**, **Table 1**). The average annual reduction in the case notification rate between 2016 and 2022 was 4.7%. Among nine provinces and the national capital, in 2022, the highest number of cases was reported by Honiara City Council (*n* = 119), the capital city, followed by Malaita province (*n* = 89) and Makira province (*n* = 34), which accounted for 71% of total cases (**Fig. 2A**). The highest rate was also reported in the same three provinces, at 126, 49 and 62 per 100 000 population, respectively (**Fig. 2B**).

[**insert** Figure 1]

[**insert** Figure 2]

**Table 1:** Number of TB case notifications and case notification rate (CNR) per 100 000 population, Solomon Islands, 2016–2022

Year	2016	2017	2018	2019	2020	2021	2022
Case notifications	402	377	391	357	321	370	343
CNR per 100 000 population	67	61	62	55	47	49	48

### Age and sex distribution

**Fig. 3:**
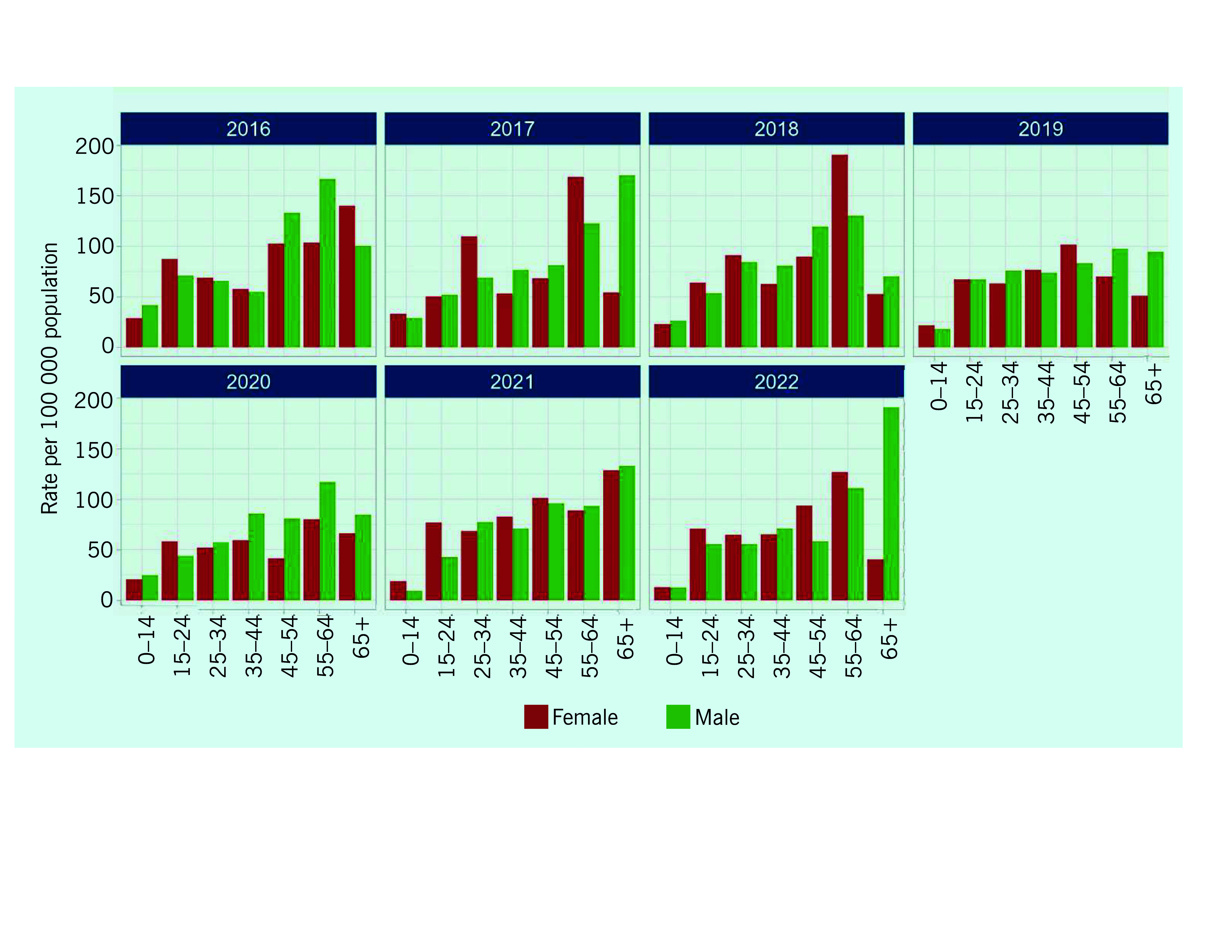
Age and sex distribution of TB notifications (new and relapse) per 100 000 population by year, Solomon Islands, 2016–2022

Among 343 new and relapse cases reported in 2022, the male-to-female ratio was 1.0 (*n* = 170 to *n* = 173). Of these 343 cases, 10% (*n* = 35) were children aged 14 years or below and 8% (*n* = 28) were adults aged 65 years or above. Between 2015 and 2022, relatively consistent trends and patterns by age group and sex were observed (**Fig. 3**). Notably, more females were diagnosed between the ages of 15–34 years, and more males were diagnosed among those aged over 55 years. The case notification rate was low for both sexes aged 14 years or below, at 9–41 cases per 100 000 population.

[**insert** Figure 3]

### Diagnosis category and type of TB

**Fig. 4:**
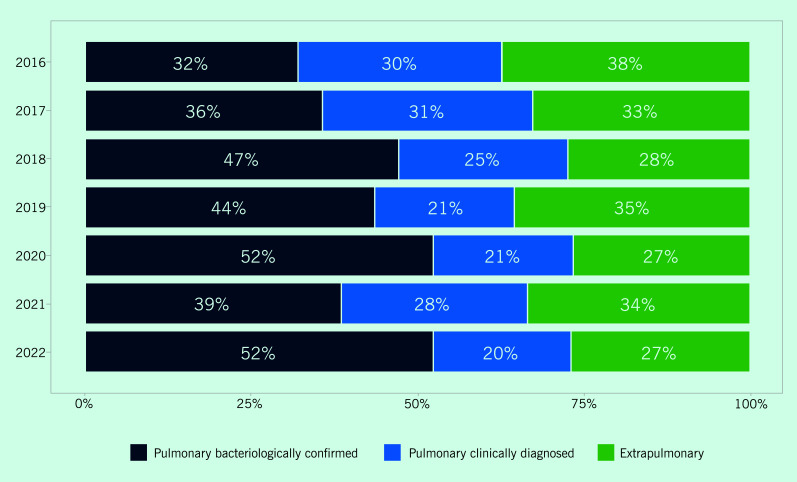
Proportion of TB notifications by year and type of diagnosis, Solomon Islands, 2016–2022

In 2022, the proportion of pulmonary bacteriologically confirmed, pulmonary clinically diagnosed and extrapulmonary TB cases was 52%, 20% and 27%, respectively (**Fig. 4**). The proportion of bacteriologically confirmed cases increased from 32% in 2016.

[**insert** Figure 4]

### Laboratory testing and drug-resistant TB

**Fig. 5:**
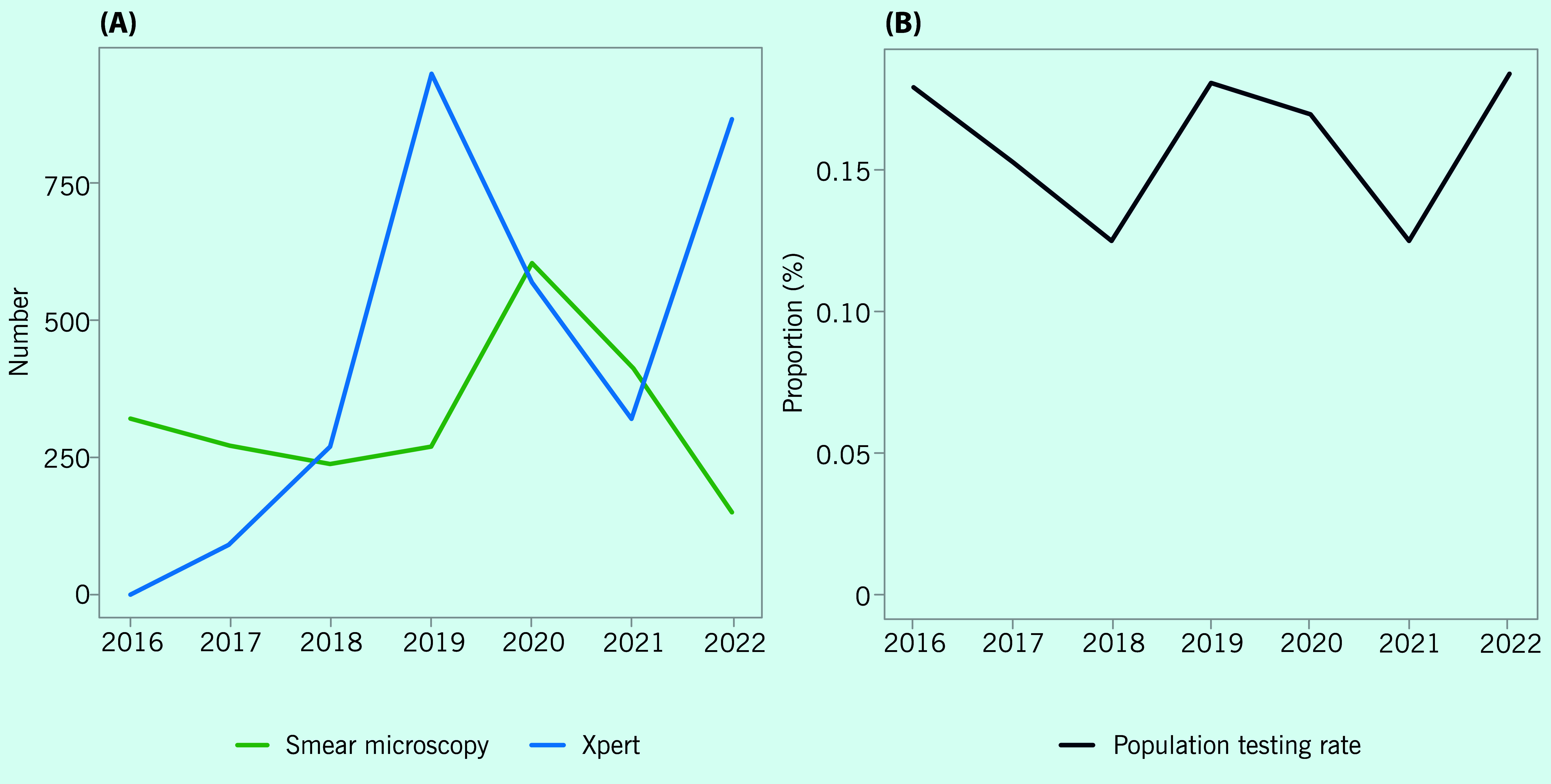
Number of presumptive TB cases on the Chronic Cough Register who were tested with smear microscopy and Xpert (A) and population testing rate (B), Solomon Islands, 2016–2022

The number of people with presumptive TB who underwent Xpert testing rapidly expanded from zero in 2016 to 951 in 2019. It then decreased to 571 in 2020 and further to 322 in 2021. However, it increased to 870 in 2022 (**Fig. 5A**). Conversely, the number of people presumed to have TB who were tested with smear microscopy increased by 124% in 2020 (*n* = 605) compared with 2019 (*n* = 270). Of those tested with Xpert between 2016 and 2022, one had rifampicin-resistant TB. The population testing rate was fairly stable, ranging from 0.13% to 0.18% between 2016 and 2022 (**Fig. 5B**).

[**insert** Figure 5]

### Treatment outcomes

**Fig. 6:**
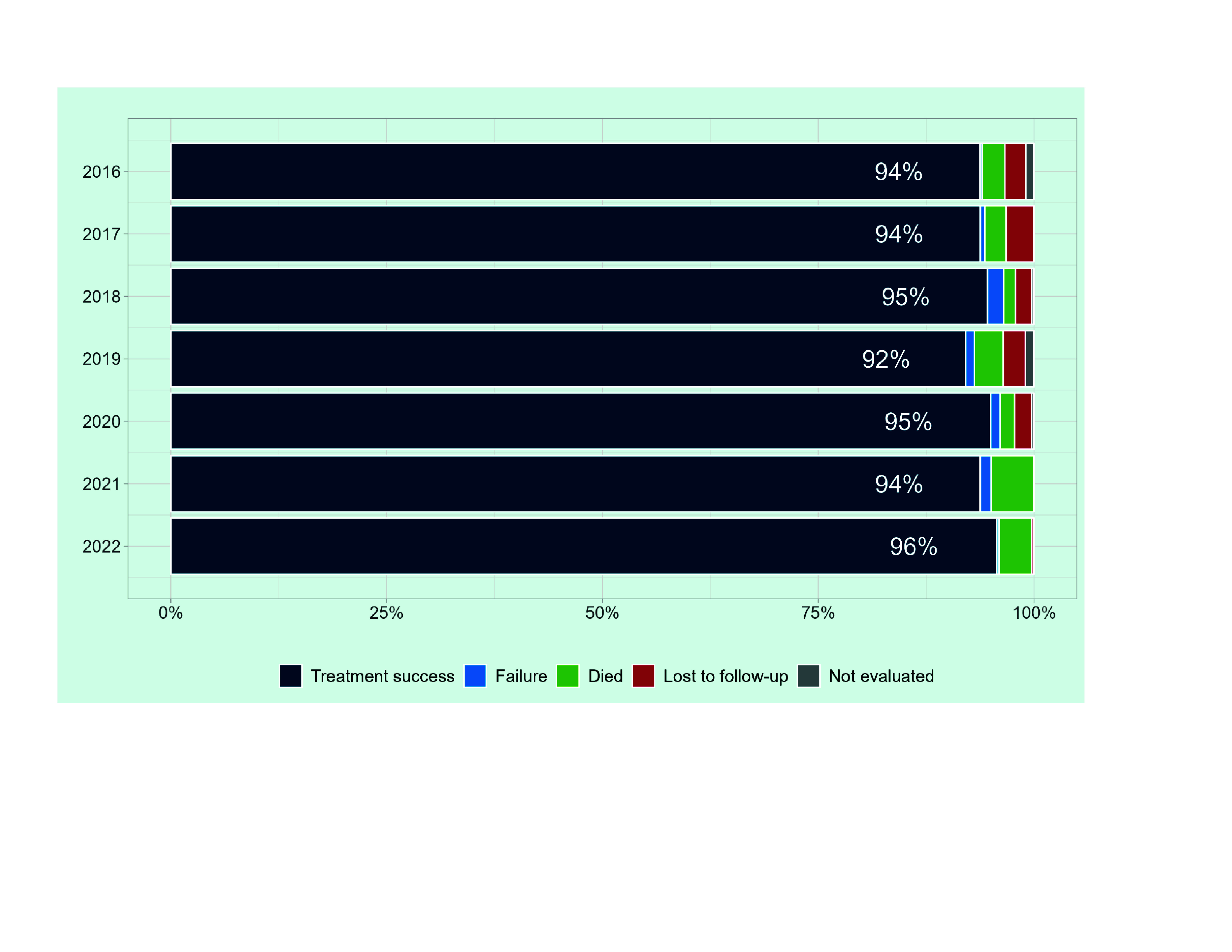
Treatment outcomes among new and relapse cases by year, Solomon Islands, 2016–2022a

**Fig. 7:**
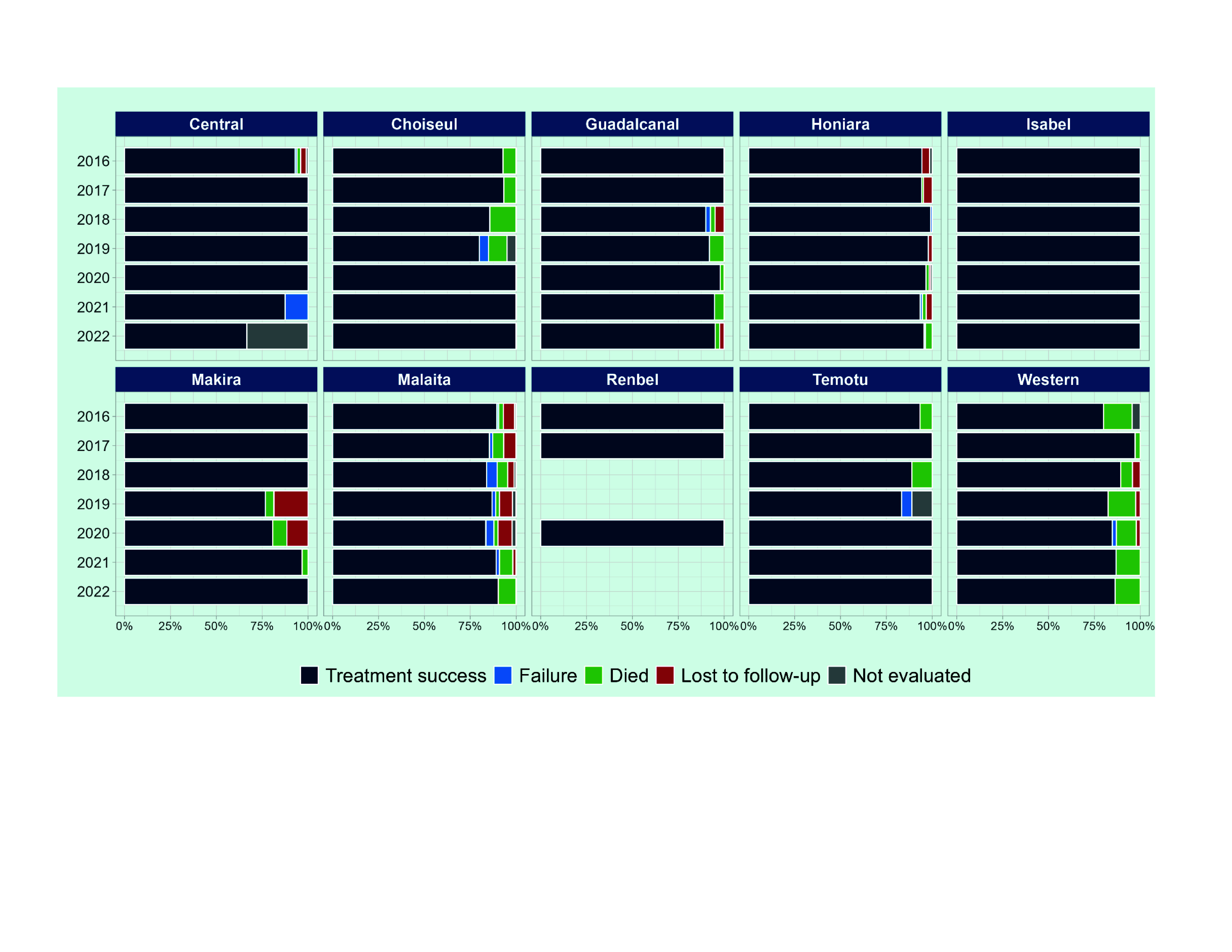
Treatment outcomes among new and relapse TB cases by province and year, Solomon Islands, 2016–2022a

Treatment success rates for new and relapse cases ranged from 92% to 96% between 2016 and 2022 (**Fig. 6**, **Table 2**). In 2022, there were no cases whose treatment outcome was not evaluated, and only one case (0.3%) lost to follow-up, with 14 deaths (4%) reported. The cure rate among bacteriologically confirmed pulmonary TB was low, at 44%, between 2016 and 2022. By province, most cases (80%) had a treatment success rate of more than 90% in 2022, except for Central province (67%) and Western province (86%) (**Fig. 7**).

[**insert** Figure 6]

[**insert** Figure 7]

**Table 2:** Treatment outcomes among new and relapse TB cases by year, Solomon Islands, 2016–2022

Year^a^	2016	2017	2018	2019	2020	2021	2022
Treatment cohort	415	402	370	391	357	321	379
Cure	118	81	44	72	50	83	70
Treatment completed	271	296	306	288	289	218	284
Failure	1	2	7	4	4	4	1
Died	11	10	5	13	6	16	14
Lost to follow-up	10	13	7	10	7	0	1
Not evaluated	4	0	1	4	1	0	0
Treatment success rate	94%	94%	95%	92%	95%	94%	96%

^a^ Year of evaluation (e.g. 2022 in the table shows the cohort in 2021).

### Indicators of collaborative TB/HIV activities

The proportion of TB cases with known HIV status – including those newly tested for HIV – increased between 2016 and 2019, from 14% to 56%, whereas it decreased in 2020 and 2021, and fell back to 14% in 2022 (**Table 3**). The number of reported TB cases coinfected with HIV was low, with two cases reported between 2016 and 2022 (0.1% [*n* = 2/2561] of total notified cases). The antiretroviral therapy (ART) coverage among coinfected cases was 100% during the same period.

**Table 3:** Known HIV status, HIV prevalence in TB patients and antiretroviral therapy (ART) coverage for TB/HIV patients by year, Solomon Islands, 2016–2022

-	Year	2016	2017	2018	2019	2020	2021	2022	Total
TB/HIV	New and relapse cases	402	377	391	357	321	370	343	2561
-	TB patients tested for HIV	57	111	109	201	123	170	49	820
	TB with HIV+	0	0	0	1	1	0	0	2
	HIV started on ART	0	0	0	1	1	0	0	2
	% of TB with HIV+	0%	0%	0%	0.5%	0.8%	0%	0%	0.2%
	Coverage of HIV screening for TB	14%	29%	28%	56%	38%	46%	14%	32%

### Indicators of collaborative TB/DM activities

Between 2016 and 2022, of the total new and relapse TB cases, 28% (*n* = 706/2561) were screened for DM. Among the individuals screened, 5% (*n* = 33/706) were diagnosed with DM. The number of patients with DM screened for TB varied by year between 2016 and 2022, ranging from 0 to 34 (**Table 4**). The proportion of patients with DM also diagnosed with TB was high, at up to 67% of those screened during the same period.

**Table 4:** People with diabetes mellitus (DM) screened for TB and people with TB screened for DM by year, Solomon Islands, 2016–2022

-	Year	2016	2017	2018	2019	2020	2021	2022	Total
TB/DM	New and relapse cases	402	377	391	357	321	370	343	2561
-	TB screened for DM	65	74	142	86	110	100	129	706
	TB with DM	2	6	6	3	6	1	9	33
	% of TB with DM	3%	8%	4%	3%	5%	1%	7%	5%
	Coverage of DM screening for TB	16%	20%	36%	24%	34%	27%	38%	28%
	DM screened for TB	15	22	28	5	11	34	0	115
	DM with TB	10	11	3	3	5	12	2	46
	% of DM with TB	67%	50%	11%	60%	45%	35%	-	40%

### Contact tracing and TPT

**Fig. 8:**
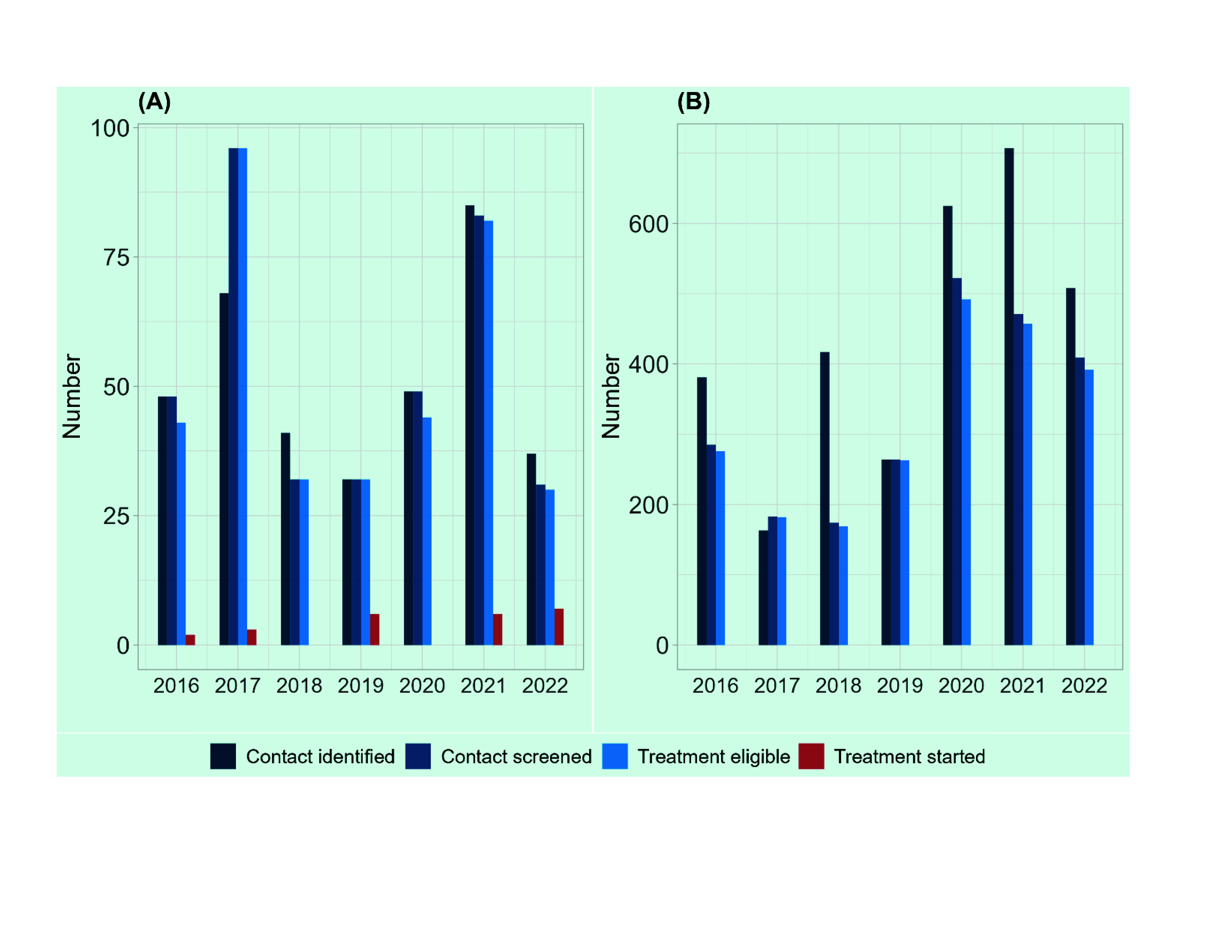
Number of TB contacts identified, screened, eligible for TB preventive treatment (TPT), and TPT started by year among household contacts aged under 5 years (A) and household contacts of all age groups (B), 2016–2022

The number of household contacts aged under 5 years varied by year, ranging from 32 in 2019 to 85 in 2021 (**Fig. 8A**). Contact tracing activities were expanded until 2021, but then reduced by 56% compared with the previous year (*n* = 37). Of the contacts identified, most (78–100%) were screened. However, among the 359 children eligible for TPT, only 7% (*n* = 24) started on TPT. The number of household contacts identified from all age groups increased (with some fluctuation), from 381 in 2016 to 707 in 2021, followed by a reduction to 508 in 2022 (**Fig. 8B**).

[**insert** Figure 8]

### Mode of care

In 2022, 97% of the patients with TB were hospitalized during the intensive phase. During the continuation phase, the proportion of patients who underwent facility-based ambulatory care decreased from 95% in 2016 to 15% in 2022, while community-based care increased from 1% in 2016 to 63% in 2022. The rest of the patients were either hospitalized (0.3%) or self-administered (22%) in 2022.

## Discussion

Our epidemiological analysis highlighted multiple programmatic achievements and challenges in Solomon Islands. The major achievements include declining case notifications despite sustained case finding efforts, consistently high treatment success rates, expansion of contact investigation initiatives and the decentralization of treatment during the continuation phase. The challenges included disruptions in TB services caused by the COVID-19 pandemic, suboptimal screening of HIV and DM among TB cases, and low uptake of TPT.

In Solomon Islands, the case notification rate consistently declined over the years of the study. Considering the sustained population testing rate observed in our analysis, the reduction of TB cases in Solomon Islands could potentially be due to the reduction in underlying TB incidence. This is supported by improving socioeconomic indicators and declining prevalence of risk factors for TB in the country, (*6*) although further epidemiological evidence is needed.

Honiara City Council reported the highest case notification rate. In general, higher density and overcrowded living conditions in populated cities contribute to transmission of TB, increasing the TB burden. However, the finding in Honiara could suggest better access to health care in the capital compared with rural provinces, indicating significant geographic disparities across provinces. The country’s geography, lack of public transport and resource constraints in health care pose a considerable challenge in population health access and cause severe financial hardship for TB-affected families. (*7*) Thus, it is critical to address potential underdetection of TB cases in rural areas.

A further important finding is the potential underdetection of specific groups, particularly elderly males. Given the global increase of male-to-female ratios in older age, there is a likelihood of underdetection among males. (*8*) This could be attributed to concerns about catastrophic costs or difference in health-seeking behaviour between males and females. (*7*, *9*) Also, there is the possibility of underreporting across the country. Some people could be identified and treated in the private sector, but the extent of the private sector’s contribution to diagnosis remains uncertain.

Our analyses demonstrated the impact of the COVID-19 outbreak on various TB activities in the country. It is likely that border controls caused interruptions to the supply of laboratory consumables including Xpert cartridges and smear reagents, which resulted in the reduction in laboratory testing in 2021. The number of Xpert tests undertaken increased to pre-pandemic levels in 2022, but case detection was affected in that year, mostly because of the reallocation of human resources, particularly during the first half of 2022 when community transmission of COVID-19 was reported. (*10*)

In recent years, facility-based ambulatory care during the continuation phase has rapidly been replaced by community-based care. This could have contributed to the increased adherence to treatment and seems to be a positive transition towards patient-centred care. However, the low cure rate among bacteriologically confirmed cases raises concerns about inadequate treatment follow-up, and evaluation during and at the end of treatment. At present, hospitalization ensures adherence to and monitoring of treatment but causes a financial burden for the affected families. To overcome this contradiction, further strengthening of community-based care by empowering communities and engaging local authorities is essential.

Our analysis suggested that screening for HIV and DM among TB patients was suboptimal. In Solomon Islands, HIV testing among TB cases might often be undervalued, given the low HIV prevalence in the general population. (*11*) Other factors (e.g. insufficient supply of test kits and people’s hesitancy around screening) might have contributed to this, which requires further investigation. Given that screening is not systematically conducted, the proportion of positive cases reported in this analysis does not represent the national prevalence; for example, the proportion of DM among screened TB cases (5% for 2016–2022) was lower than the estimated DM prevalence among the general population aged 20–79 (19.8% in 2021). (*12*) Although the number of DM patients screened for TB was limited, the high proportion of TB cases detected demonstrated the effectiveness of screening for TB among DM patients in this context. They might be prescreened by TB symptoms or other TB risk factors.

Although contact investigation activities were expanded, the number of people who started on TPT was low, representing a missed opportunity to intensify TB prevention. Anecdotal evidence suggested that clinicians were hesitant to prescribe TPT owing to uncertainty about eligibility, and that this was coupled with lack of acceptance by eligible populations. To counteract this situation, it is necessary to train the health-care providers and provide education to eligible patients. At the same time, systematic recording and reporting of TPT implementation from treatment initiation to completion is required to inform corrective actions.

Our analysis was limited by the relatively small number of cases included because of the small population size. This makes it challenging to determine definitive trends and patterns, especially in age- and sex-disaggregated data analysis and province-level analysis. Additionally, the quality of the surveillance data was heavily dependent on local facility and staff capacity. Manual data entry using paper-based records and reports might have led to inconsistencies in some of the data reported. In particular, the data from the Chronic Cough Register that were used to describe the trends of laboratory testing did not fully match the data from the laboratory records.

Despite these limitations, our analyses provided a comprehensive insight into TB epidemiology and programmatic performance in Solomon Islands. For the country to stay on track to achieve the goal of ending TB by 2035, it is imperative to address the identified challenges through the efficient use of existing resources. Moreover, concerted national efforts to build resilient health systems, expand health service coverage and address social determinants of health are crucial in advancing the national TB response in Solomon Islands.
